# The Darkest Place Is under the Candlestick-Healthy Urogenital Tract as a Source of Worldwide Disseminated Extraintestinal Pathogenic *Escherichia coli* Lineages

**DOI:** 10.3390/microorganisms10010027

**Published:** 2021-12-24

**Authors:** Magdalena Ksiezarek, Ângela Novais, Luísa Peixe

**Affiliations:** 1 UCIBIO–Applied Molecular Biosciences Unit, REQUIMTE, Laboratory of Microbiology, Department of Biological Sciences, Faculty of Pharmacy, University of Porto, 4050-313 Porto, Portugal; mag.ksiezarek@gmail.com (M.K.); angelasilvanovais@gmail.com (Â.N.); 2 Associate Laboratory i4HB-Institute for Health and Bioeconomy, Faculty of Pharmacy, University of Porto, 4050-313 Porto, Portugal

**Keywords:** vaginal microbiome, voided urine, urinary tract microbiome, uropathogens, ExPEC, ST131

## Abstract

Since the discovery of the urinary microbiome, including the identification of *Escherichia coli* in healthy hosts, its involvement in UTI development has been a subject of high interest. We explored the population diversity and antimicrobial resistance of *E. coli* (*n* = 22) in the urogenital microbiome of ten asymptomatic women (representing 50% of the sample tested). We evaluated their genomic relationship with extraintestinal pathogenic *E. coli* (ExPEC) strains from healthy and diseased hosts, including the ST131 lineage. *E. coli* prevalence was higher in vaginal samples than in urine samples, and occasionally different lineages were observed in the same individual. Furthermore, B2 was the most frequent phylogenetic group, with the most strains classified as ExPEC. Resistance to antibiotics of therapeutic relevance (e.g., amoxicillin-clavulanate conferred by bla_TEM-30_) was observed in ExPEC widespread lineages sequence types (ST) 127, ST131, and ST73 and ST95 clonal complexes. Phylogenomics of ST131 and other ExPEC lineages revealed close relatedness with strains from gastrointestinal tract and diseased host. These findings demonstrate that healthy urogenital microbiome is a source of potentially pathogenic and antibiotic resistant *E. coli* strains, including those causing UTI, e.g., ST131. Importantly, diverse *E. coli* lineages can be observed per individual and urogenital sample type which is relevant for future studies screening for this uropathogen.

## 1. Introduction

Urinary tract infections (UTIs) are reported to be one of the most common infections worldwide, and occur more frequently in women than in men due to anatomical differences, and its incidence increases with age or sexual activity [1,2,3,4]. Although several bacterial species are reported as causative agents of UTIs (e.g., *Escherichia coli*, *Klebsiella* sp., *Proteus* sp., *Staphylococcus saprophyticus*), *E. coli* is responsible for the majority (up to 75%) of both uncomplicated and complicated UTIs [2,5,6,7].

Extraintestinal pathogenic *E. coli* (ExPEC) strains are most frequently responsible for these infections, with this pathotype associated with genetic characteristics that seem to favor the pathogenicity of particular strains, but also *E. coli* persistence in the urinary tract [8,9,10]. Indeed, most UTIs are caused by a subset of ExPEC strains [e.g., sequence type (ST) 131, ST95, ST69, ST73, ST127, ST12] that are highly disseminated across different continents and populations and represent great clinical challenges not only because they are often resistant to the main therapeutic choices but also because of their extended reservoirs (human and animal gastrointestinal tract and the environment) [11,12,13,14,15,16].

To date, multiple studies have evaluated putative human-related reservoirs of ExPEC strains causing UTI. The human gut or vaginal colonization with certain strains of *E. coli* have been considered risk factors for developing UTI [17,18,19,20,21]. Additionally, the existence of intracellular bacterial communities or quiescent intracellular reservoirs that may silently persist within the epithelial cells of the urogenital tract also contribute to recurrent UTI (rUTI) development [7,22].

On the other hand, the collapse of the urine sterility dogma after the identification of the female urogenital microbiome (FUM) [23] and the urinary microbiome [24], raised the possibility of the urinary/urogenital tract being a reservoir of UTI pathogens [25,26]. In fact, studies analyzing FUM composition unveiled the presence of uropathogenic bacterial species, including *E. coli*, in the healthy urinary tract [27,28]. Moreover, a high inter-individual variability in the relative abundance of *E. coli* (varying from 10 to >10^5^ CFU/mL) was reported in the healthy population [27,29,30]. Thus, since *E. coli* is the most frequent pathogen in causing UTI and because it is part of the healthy FUM, occasionally in high relative abundance, it is plausible that the urinary tract itself is a source of ExPEC lineages causing UTIs.

Until now, FUM characterization has focused mostly on the taxonomic profiling at the genus and species level, whereas characterization at the strain level is scarce. Thomas-White and co-workers [28] revealed the urinary and vaginal interconnection for potentially pathogenic *E. coli*. However, no data on virulence potential or antimicrobial resistance profiles of *E. coli* strains were provided. Strain level characterization from healthy hosts was only performed recently in a study from Garretto et al., that aimed at establishing an association between the genomic content of urinary strains and the presence of lower urinary tract symptoms [31]. The authors did not find any specific signatures on the microbiome composition, gene content or *E. coli* abundance that could predict UTI status, and only six urinary strains were obtained from asymptomatic women [31]. Thus, the nature and diversity of *E. coli* strains from the whole urogenital tract (urinary and vaginal) of healthy women are still practically unexplored.

In our study, we aimed to characterize the prevalence, strain diversity and genetic features (antimicrobial resistance and virulence) of *E. coli* isolated from both urine and vaginal samples of healthy asymptomatic women. Furthermore, we provided an in-depth phylogenomic analysis of the phylogenetic group B2 ExPEC strains.

## 2. Materials and Methods

### 2.1. Study Design and Sample Description

*E. coli* strains were isolated in the framework of a study on healthy Female Urogenital Microbiome conducted at Faculty of Pharmacy, University of Porto, Porto, Portugal (2016–2019) [30]. All women provided informed written consent for participation in the study. The study was developed according to the Helsinki Declaration principles and the protocol was submitted and approved by the Ethical Commission of Faculty of Pharmacy, University of Porto (Ethical Committee approval number 32-09-2017). This study included isolates obtained from 20 healthy women (aged 24–57) who were asymptomatic for any urinary disorder and that declared to be in good health condition, from which urogenital samples (30 voided urine and 30 vaginal swabs) were analyzed (10 women provided samples twice [30]). A list of donors, samples and isolates is presented in Supplementary Table S1.

### 2.2. Identification of E. coli Isolates

All 60 samples were analyzed based on a previously published extended culturomic protocol [30]. When colonies with a morphology compatible with *E. coli* were observed, an average of 3 isolates/samples (up to 7 colonies/sample; proportionally to the bacterial load) were isolated.

Isolates were preliminarily identified by MALDI-TOF MS (VITEK MS, bioMérieux, France), and further confirmed by the amplification of the *malB* gene [32]. A total of 50 *E. coli* isolates were identified and stored, 22 of which originated from the urine of healthy donors (1–7/sample) and the remaining 28 were detected from vaginal swabs of healthy donors (1–5/sample) (Supplementary Table S1).

### 2.3. Characterization of E. coli Population

The identification of *E. coli* phylogenetic groups and virulence gene profiling were performed on all *E. coli* isolates so as to discard overrepresented genotypes per sample type (urinary, vaginal). *E. coli* phylogenetic groups were identified according to the method proposed by Clermont et al. [33]. The presence of 41 virulence factors (VFs) associated with ExPEC pathotype including adhesins, toxins, siderophores, capsule type, protectins, invasins and miscellaneous was screened by PCR (Supplementary Table S2). Detection of *hlyA* operon duplication was performed by PCR, as previously described [34] (Supplementary Table S2). The strains were classified as putative ExPEC if fulfilled the following criteria: presence of >2 amongst *papAH* and/or *papC*, *sfa*/*focDE*, *afa*/*draBC*, *kpsM* II and *iutA* [35].

Based on this strain profiling strategy, we selected 10 representative urinary and 12 representative vaginal *E. coli* strains, which are described in detail in Table 1. To characterize the representative strains of the urogenital population, pulsed-field gel electrophoresis (PFGE) was used to identify identical strains detected in both urine and vaginal samples from the same donor. The standard protocol for PFGE adapted from Gautom et al. [36] was used, with 1.6% chromosomal grade agarose (SeaKem^®^ Gold Agarose) for plugs preparation, 1.2% gel with pulse-field certified agarose (SeaKem^®^ Gold Agarose) for PFGE run, genomic DNA digested with 20U *Xba* I and CHEF DNA Size Standard–Lambda Ladder, which was used as a size marker. The running conditions included an initial switch time of 2.2 s, a final switch time of 63.8 s and a run time of 19 h with 6 V/cm and an angle of 120.

This procedure narrowed down our sample to 16 unique strains representing unique genotypic profiles from the urogenital *E. coli* population of 10 healthy women. In vitro antibiotic susceptibility testing was evaluated using a disk diffusion for 21 antibiotics (diverse β-lactams, fluoroquinolones, aminoglycosides, tetracyclines, fosfomycin, nitrofurantoin, trimethoprim, sulfamethoxazole-trimethoprim) according to EUCAST guidelines (www.eucast.org, accessed on 12 December 2019).

### 2.4. Whole Genome Sequencing

Considering the high proportion of B2 *E. coli* strains (50%, 8/16) and the identification of typical ExPEC pathotype features amongst them, 8 B2 strains were characterized using whole genome sequencing (WGS) and comparative genomics approaches, as described below.

Genomic DNA (*n* = 4 urine, *n* = 4 vaginal) was extracted (Wizard Genomic DNA Purification Kit, PROMEGA) and sequenced by Illumina NovaSeq 2 × 150 nt. Reads were trimmed by Trimmomatic [37] version 0.39 and the quality of reads was checked by FastQC version 0.11.9 (http://www.bioinformatics.babraham.ac.uk/projects/fastqc/, accessed on 2 February 2021). De novo assembly was performed by SPAdes [38] version 3.13.0 and the quality of the assembly was checked by Quast [39] version 5.0.2. Annotations of the draft genomes were provided by the NCBI Prokaryotic Genome Annotation Pipeline, and additionally by Prokka [40] version 1.14.6.

The Whole Genome Shotgun project has been deposited at DDBJ/ENA/GenBank under the BioProject accession number PRJNA548360. Accession numbers to WGS submission and the BioSample for each strain are available in Supplementary Table S3.

Characterization of strains including serotype, *E. coli* phylogenetic group and multilocus sequence typing (MLST, Achtman scheme) was assessed using available in silico tools (http://www.genomicepidemiology.org/, accessed on 4 February 2021; http://clermontyping.iame-research.center/, accessed on 4 February 2021). The virulence profile of each strain previously defined by PCR screening was verified and extended to 49 putative virulence genes using in-house database (https://github.com/magksi/E.coli_VF_characterization, released on 20 December 2021) and ncbi-blast-2.8.1+ package (https://ftp.ncbi.nlm.nih.gov/blast/executables/blast+/, accessed on 7 May 2019). Antimicrobial resistance genes were identified using ResFinder 4.1 (https://cge.cbs.dtu.dk/services/ResFinder/, accessed on 4 February 2021), plasmid replicon sequences by PlasmidFinder 2.1 (https://cge.cbs.dtu.dk/services/PlasmidFinder/, accessed on 4 February 2021) and *fimH* typing using FimTyper 1.0 (https://cge.cbs.dtu.dk/services/FimTyper/, accessed on 4 February 2021).

### 2.5. Comparative Genomics of B2 E. coli Strains

We extracted 19,668 *E. coli* assemblies from the NCBI Assembly database (assessed at 14 July 2020), including 1266 complete genomes and 18,402 draft genomes (7432 scaffolds and 10,970 contigs). To enlarge the sample of urinary isolates from both healthy and diseased women, we included 66 *E. coli* assemblies of urinary origin, published in September 2020 by Garretto et al. [31] and 3 B2 *E. coli* genomes recovered from the urine of 3 women with a history of recurrent UTI from our collection (Supplementary Table S3).

A total of 19,737 assemblies were subjected to mlst 2.19.0 pipeline [41] (https://github.com/tseemann/mlst, accessed on 10 February 2021) to access STs of public genomes. Genomes (*n* = 1084) that represented the same STs as those detected in our collection were extracted. Only the genomes from humans and those with available metadata on host health status and isolation source were considered. Following these criteria, we grouped isolates according to host health status (healthy-H or diseased-D) and origin (urogenital tract, gastrointestinal tract or other isolation source). Additionally, complete genomes of well-known reference strains for certain ST were included in the analysis.

### 2.6. Single Nucleotide Polymorphisms Analysis

The genomes were subjected to a snippy (version 4.4.0, https://github.com/tseemann/snippy, accessed on 12 February 2021), using snippy-multi and mapping to appropriate reference genome (stated in description of the figures). A core genome and whole genome SNPs alignments generated by snippy were used for Gubbins pipeline (Genealogies Unbiased By recomBinations In Nucleotide Sequences) [42] version 2.4.1 to remove recombinant regions. The SNP matrices from core genome and whole genome alignment for comparison were generated using snp-dist version 0.7.0 (https://github.com/tseemann/snp-dists, accessed on 12 February 2021). The resulting SNP-based alignments were used to reconstruct the phylogeny for each ST using RAxML, with the appropriate reference. The obtained phylogenetic trees were represented using the Interactive Tree of Life (iTOL, https://itol.embl.de, accessed on 15 February 2021).

### 2.7. Accessory Genome and Ordination-Based Analysis

A total of 520 ST131 *E. coli* genomes were annotated with prokka version 1.14.6, and a pangenome analysis was performed with Roary [43] version 3.13.0 with the default criteria for proteins identity of 95%. The accessory genome included genes that were present in <99% of the genomes. The data were reshaped and cleaned with tidyverse [44] package version 1.3.0. Further analyses, including non-metric multidimensional scaling (NMDS), and a calculation of the stress value was performed in R [45] version 4.0.3 with package vegan [46] version 2.5.7 and ggplot2 [47] version 3.3.5.

### 2.8. Statistical Analysis

Continuous variables were interpreted based on the descriptive statistics. Welch Two Sample *t*-test in R [45] version 3.6.2 was used to access significance of phylogenetic groups and VF distribution between the different sample types. Heatmap and clustering of the isolates based on VFs presence/absence was performed with Pheatmap package version 1.0.12 (https://github.com/raivokolde/pheatmap, accessed on 1 March 2021), with clustering based on Euclidean distance.

## 3. Results

### 3.1. Frequency and Diversity of E. coli in Urinary and Vaginal Microbiome

*E. coli* was identified in 30% of the urine samples (9/30) and 37% of the vaginal samples (11/30) (Supplementary Table S1). The bacterial load varied from 1 × 10 CFU/mL – 1 × 10^7^ CFU/mL, with a median of 1 × 10 CFU/mL. Overall, *E. coli* was identified in 50% (10/20) of the total cohort of asymptomatic women.

A set of 10 urinary and 12 vaginal representative *E. coli* strains is presented in Table 1. All but one of the B2 strains were classified as ExPEC. Furthermore, two isolates belonging to phylogenetic group F (c1Ub_48 and c10Ua_105) were also considered ExPEC (Table 1). Interestingly, two donors carried different *E. coli* strains in either urine or vaginal samples and two donors had different *E. coli* strains in the same sample type.

Although there was no significant association between the phylogenetic groups’ distribution or VF profiles and the origin (urine or vaginal) of the sample, *E. coli* phylogenetic group B2 were more frequently detected in vagina (58%) compared to the urinary isolates (40%). Other phylogenetic groups (F, D and A) were variably found in either urine and vagina samples, while C and E were detected in only one sample type (Table 1 and Table 2).

Certain VFs were highly prevalent in urinary and vaginal isolates (Table 2) such as adhesins *fimH* (90% and 92%, respectively), *matB* (80% and 83%, respectively) or *ompT* (80% and 83%, respectively). Several toxins and siderophores were detected in strains from both niches, while invasins were only detected in vaginal isolates. We also found that many urinary and vaginal strains possess *kpsMT* II type (50% and 58%, respectively).

### 3.2. Virulence Profile Characterization of Urogenital E. coli

A detailed characterization of virulence gene profiles was performed on representative isolates per donor, independently on the sample type. For this purpose, vaginal isolates that presented identical PFGE profiles to urinary isolates (Supplementary Figure S1, Table 1) from the same individual and phylogenetic group were discarded, leaving a total of 16 representative urogenital strains from healthy women.

**Figure 1:**
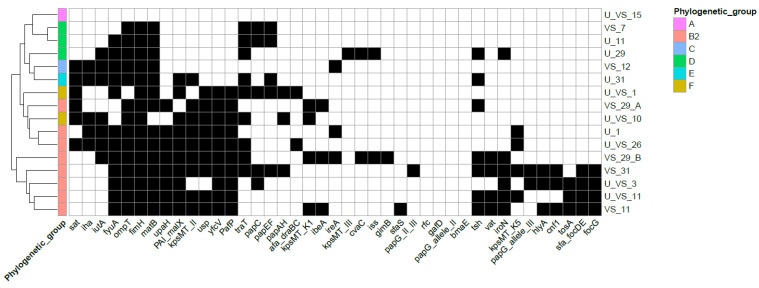
Heatmap representing presence/absence matrix of putative virulence genes among urogenital *E. coli* from different phylogenetic groups. The dendrogram in the left represents clustering of isolates based on Euclidean distance. Presence or absence of virulence genes is represented as black or white squares, respectively. The right hand side of the figure contains the list of unique *E. coli* profiles coded with U (for urinary) or VS (for vaginal swab) for origin and a number for identification of donor, according to Table 1.

A hierarchical clustering analysis was performed to assess strain similarity based on VF profiles (Figure 1). Isolates were grouped into the following three primary clusters: (i) representing strains from B2 phylogenetic group (*n* = 5) enriched in *hlyA*, *cnf*1, *tsh*, *vat* and *iroN*; (ii) strains from B2 (*n* = 3) and F (*n* = 2) phylogenetic group with *sat* and *iutA* (without adhesins and previously mentioned genes) and (iii) strains belonging to other phylogenetic groups (*n* = 6), less enriched in VF (without *yfcB*, *usp*, *upaH*, *pafP*). One strain belonging to phylogenetic group A did not possess any virulence gene screened (Figure 1).

### 3.3. Genomic Background and Antimicrobial Resistance of B2 E. coli Strains

The genetic features of the eight B2 ExPEC strains subjected to whole genome sequencing (WGS) are presented in Table 1, and characteristics of draft genomes assemblies and accession numbers are available in Supplementary Table S3. Remarkably, some of these B2 strains were identified as pandemic *E. coli* lineages such as ST131, ST127, clonal complex 73 or 95. Others were identified as ST452, ST569, ST681 and ST998 (Table 1). They represented various serogroups, including O2, O6, O8, O25, O46 and O81 (Table 1). Additionally, we performed an MLST analysis of the B2 strains from asymptomatic women identified in the previous study by Garretto et al. [31]. Remarkably, those strains also belonged to intercontinental STs including ST95 (*n* = 3), ST73 (*n* = 1), ST12 (*n* = 1) and ST1193 (*n* = 1).

**Figure 2:**
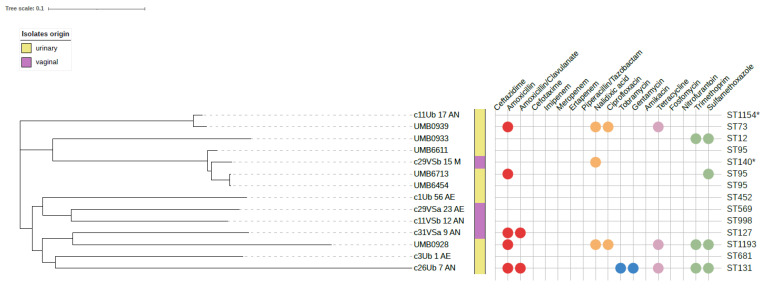
Phylogenetic tree representing core genome SNPs alignment of 14 urogenital *E. coli* strains isolated from asymptomatic women (8 from our study and 6 from Garretto et al.). The alignment was performed using *E. coli* strain UTI89 as a reference. The tree is unrooted. Colored balls represent the resistance phenotype/genotype to a given antibiotic (colors by antibiotic class). Resistance of the strains originated from this study was characterized by phenotypic and genotypic methods, while strains from Garretto et al. were characterized genotypically. Single locus variants (SLV) are represented with an asterisk (see Table 1).

We performed a core genome single nucleotide polymorphism (SNP) alignment of 14 B2 genomes isolated from asymptomatic women (8 from our study and 6 from Garretto et al. [31]), as shown in Figure 2. According to this phylogenetic tree, the genomes were substantially different and presented an average of ~28,000 SNP differences. The most related strains belonged to the ST95 clonal complex (UMB6611, c29VSb_15_M, UMB6713, UMB6454) which has ~180–4250 SNP differences, followed by the ST73 clonal complex (c11Ub_17_AN and UMB0939) which differed by 2353 SNP.

Overall, 7/14 strains were resistant to at least one antibiotic, including antibiotics used to treat UTI (Figure 2). The ST131 (c26Ub_7_AN) and ST1193 (UMB0928) strains revealed multidrug resistance phenotypes (resistance to 7 and 6 antibiotics from 4 classes, respectively), including to critical antibiotics for UTI treatment (trimethoprim/sulfamethoxazole and amoxicillin-clavulanate) [48]. Additionally, ST12, ST73, ST95, ST140 (SLV 95) and ST127 strains were resistant to amoxicillin-clavulanate, nalidixic acid, ciprofloxacin, trimethoprim/sulfamethoxazole and/or tetracycline (Figure 2). The detection of the acquired resistance genes in our genomes was compatible with phenotypic characterization of resistance: (i) *bla*_TEM-30_, in strains resistant to amoxicillin-clavulanic acid combination; (ii) *tet*(A) for tetracycline resistant strain; (iii) *aac*(3)-*IId*, *sul*1 or *dfrA*12 conferring resistance to aminoglycosides or trimethoprim (Supplementary Table S3). We also observed mutations in *gyrA* (S83L) for isolate c29VSb_15M, conferring resistance to fluoroquinolones (Figure 2, Supplementary Table S3).

Screening of plasmid replicon sequences in our eight isolates revealed that no known replicon sequences were detected for four of the strains (ST569, ST681, ST998, ST1154), whereas, in the other B2 strains, several replicon types were detected, mostly Col156 and IncFIB (AP001918) (Supplementary Table S3).

### 3.4. Phylogenomics of Urogenital ST131 E. coli from Healthy Urinary Microbiome

We performed a whole genome SNP phylogeny analysis in relation to the origin and health status of the ST131 *E. coli* strain isolated from asymptomatic woman comparatively with representative ST131 genomes from the NCBI public database.

In a total of 520 ST131 genomes for which metadata was available, most were obtained from human infections (D; *n* = 422 strains) and much less frequently from healthy carriers (H; *n* = 98 strains). They were identified mainly in wounds or pus, lung and sputum, blood or other body fluids (other; *n* = 242 strains), followed by strains isolated from the urinary (*n* = 179 strain) and gastrointestinal tract (*n* = 99 strains). While isolates obtained from the gastrointestinal tract originated mostly from healthy humans (H = 96, D = 3), isolates from the urinary tract were almost exclusively associated with urinary tract infections and only one genome (from this study) originated from healthy host (H = 1, D = 178). Isolates from other human niches were mostly isolated from the diseased host (H = 1, D = 241), e.g., bacteremia, sepsis, pneumonia.

**Figure 3:**
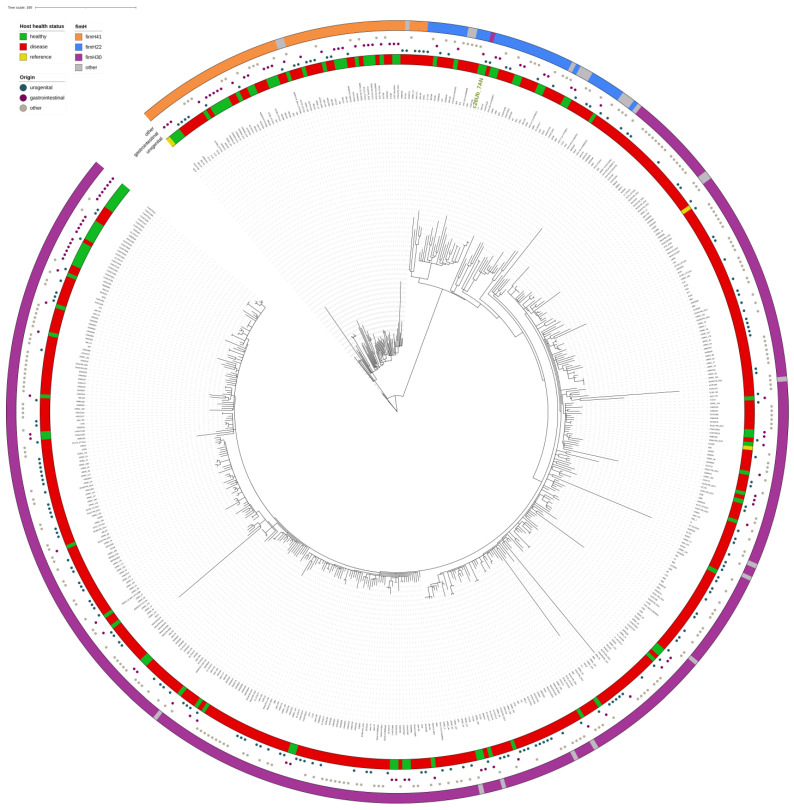
Core genome SNPs phylogenetic tree of 520 ST131 *E. coli* genomes of human origin. The phylogenetic tree includes 3 ST131 reference strains, i.e., SE15, JJ1886, EC958. The alignment was performed using *E. coli* strain EC958 as a reference and *E. coli* SE15 was used as an outgroup. The metadata including host health status, strain origin and *fimH* type are incorporated in the tree, as explained in the legend. The identifier of the strain from our urogenital collection is marked in green.

The core genome phylogenetic tree was congruent with previous ST131 phylogenetic inferences and establishes the clustering of genomes in three clades corresponding to clade A (*n* = 67), clade B (*n* = 50) and clade C (*n* = 406) (Figure 3). Our ST131 strain from healthy woman (c26Ub_7_AN) clustered in clade B isolates despite carrying *fimH*30 and showed similarity to other clade B genomes from gastrointestinal tract colonizers (1111 SNPs) or infections (1157 SNPs). Interestingly, to date, this genome represents the unique ST131 from the urogenital tract of a healthy individual.

**Figure 4:**
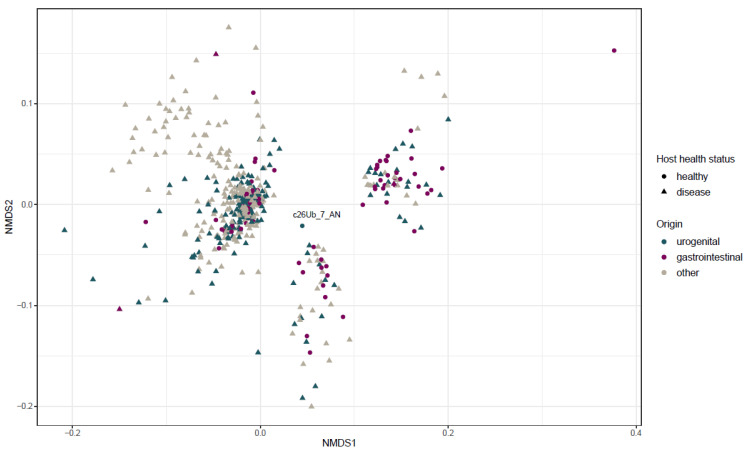
Non-metric multidimensional scaling (NMDS) based on 28,542 genes matrix (accessory genome) extracted from 520 ST131 *E. coli* genomes. Metadata layers of origin and host health status are incorporated in the figure. Stress value for this ordination is 0.18.

We then examined strains similarity according to their accessory genome (Figure 4). The NMDS ordination based on a 28,542 accessory gene matrix demonstrated three different clusters that are likely to correspond to clades A, B and C (from the right to the left, respectively; Figure 4), irrespective of host health status or the isolate’s origin. The urinary strain c26Ub_7_AN isolated from healthy woman was identified in the proximity of two clusters.

Overall, genomes from different niches and disease statuses were closely related and randomly distributed in the phylogeny, often with less than 50 SNPs among them. The SNPs matrix for the ST131 core-genome-based phylogenetic tree is available in Supplementary Table S4.

### 3.5. Phylogenomic Analysis of Other B2 E. coli Strains

A whole genome comparison was performed between our B2 non-ST131 isolates and the available genomes from the same ST on public databases. In agreement with the available literature, ST95 (*n* = 272) including ST140 (SLV 95) and ST73 (*n* = 217) including ST1154 (SLV 73) are the second and third most represented clonal groups (Supplementary Figures S2 and S3, respectively). SNP-based phylogenetic trees containing our ST140 (ST95 clonal complex), and ST1154 (ST73 clonal complex) urogenital *E. coli* isolates also evidenced a high similarity between strains from different host status and origin, often showing <1000 SNPs differences. The same was observed for the clone belonging to ST127 (*n* = 66), which was closely related to genomes from isolates causing UTI and/or isolated from other niche (<300 SNP differences, Supplementary Figure S4).

**Figure 5:**
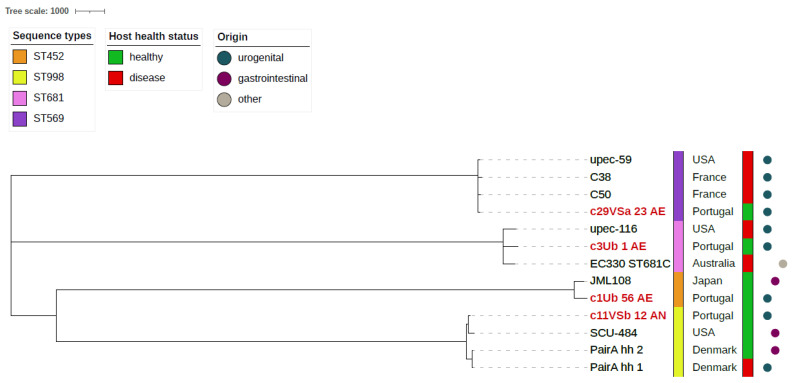
Whole genome SNPs phylogenetic tree of 4 infrequent *E. coli* STs (ST452, ST569, ST681, ST998) including 4 from our urogenital collection (marked in red) and 9 genomes retrieved from NCBI database. The alignment was performed using *E. coli* strain 536 as a reference. The tree is unrooted. Information on ST, health status of the host and origin of the strains is incorporated in the tree.

The remaining four STs (ST452, ST569, ST681, ST998) were much less represented in the NCBI database (uncommon STs). However, a comparative genomic analysis also showed, a high similarity (390–9452 SNPs) to genomes deposited from other sources (Figure 5). Interestingly, the corresponding strains were isolated in different continents.

## 4. Discussion

In this study, we provided a detailed strain-level analysis of the *E. coli* isolates identified in the female urogenital microbiome, revealing that the urogenital tract of healthy women is an additional reservoir of pandemic ExPEC clones with the potential to cause UTI. Furthermore, we found an ST131 strain inhabiting the urogenital tract of healthy woman with a similar genetic background to those causing UTI or colonizing the gastrointestinal tract of humans.

*E. coli* was often found both in the urine (30%) and vagina (37%) of healthy women, the latter revealing a prevalence of slightly higher than that reported in previous studies [20,49]. Our data further supports that there is an interconnection between these two niches [28] in the same individual since the urine and vagina often share identical strains. However, in some samples, we also found variability in the number and type of *E. coli* strains identified in each of those niches, highlighting possible limitations in studies evaluating only one of the two locations for a risk assessment of UTI acquisition. Furthermore, our data also highlights that strain level characterization is relevant to accurately evaluate the contribution of these niches as reservoirs of strains with the potential to contribute to the persistence and/or cause UTI.

We described a higher *E. coli* diversity in the urogenital microbiome of healthy women (phylogenetic groups A, B2, D, E, F) than that reported in the study from Garretto et al., where only B2 *E. coli* strains (*n* = 6) were identified in asymptomatic women [31].

We demonstrated that strains from healthy urinary microbiome belong to serogroups known to be commonly involved in UTI (e.g., O2, O6, O25) [13,50,51]. Remarkably, the identification of ExPEC (*n* = 7 B2; *n* = 2 F) strains in healthy women was also observed, enriched in putative virulence factors known to favor colonization and/or the invasion of epithelial cells and the development of UTI [6]. We found that the virulence gene profiles detected in the ExPEC isolates from healthy women were identical to those described in clinical isolates [16]. All other non-ExPEC strains, except one phylogroup A *E. coli*, also possessed several adhesins, siderophores and other putative virulence genes, likely supporting their adaptation and survival in the urogenital tract. Thus, our study provides further evidence that the urogenital tract carries strains with pathogenic potential.

Remarkably, half of our B2 strains from asymptomatic women belonged to worldwide disseminated *E. coli* lineages (ST73 complex, ST95 complex, ST131, ST127), that have caused infections in humans [13,15,17,18]. Our phylogenomic analysis revealed close relatedness with publicly available genomes regardless of human origin (host status or infection site), further demonstrating the wide circulation of well-adapted and potentially pathogenic clones between different individuals and an interconnection between the urogenital and the gastrointestinal tract, which seems to be the most probable source of these strains.

The identification of strains, including ST131, in the urogenital tract of healthy women with phenotypes resistant to antibiotic classes used to treat UTI (amoxicillin-clavulanate, ciprofloxacin, trimethoprim/sulfamethoxazole; Figure 2) represents an additional concern due to the risk of treatment failure. Moreover, antibiotic-resistant strains in the microbiome constitute a reservoir of transferable antimicrobial resistance genes that can be shared with other strains/species by horizontal gene transfer [52,53]. Thus, the possibility of enriching antibiotic-resistant bacterial species (e.g., *E. coli*, *Citrobacter koseri*, *Klebsiella pneumoniae*) [29,30] in the urogenital microbiome can have direct implications on human health in future.

The identification of ST131 *E. coli* in the urine of healthy woman challenges the current understanding of the ecology of this pandemic clone. We demonstrate, for the first time, that the healthy urogenital microbiome can itself act as a reservoir of putative pathogenic and antibiotic resistant ST131 strains. We further highlighted that closely related isolates from different clades are identified as colonizers of the gastrointestinal or urogenital tracts of healthy people and can cause disease [54,55], and that the accessory genome structures those clades irrespective of niche origin. Interestingly, our ST131 strain that was isolated from a healthy woman (c26Ub_7) belongs to clade B, despite having *fimH*30. The detection of the *fimH*30 allele in clade B isolates is uncommon, but these recombination events have been occasionally observed in other collections [11,21]. Available data on the prevalence of ST131 subclades is largely biased by human clinical isolates, and clade C in particular [56,57,58]. However, there is evidence that non-human sources are underestimated and there is a specialization for each clade, including a foodborne origin for clade B-*fimH*22 [59] and wastewater for clade A-*fimH*41 [58], suggesting those as additional possible sources of acquisition.

Besides ST131, other intercontinental and/or emerging B2 *E. coli* clones were found in the urogenital microbiome of healthy women (i.e., ST95 complex, ST73 complex, ST127, ST12 and ST1193) [21,60,61]. Overall, these data suggest that the healthy urogenital microbiome is a source of particularly widespread (e.g., food, domestic animals, environment) *E. coli* lineages that are frequently responsible for UTI [62,63].

Much less data are available for uncommon and antibiotic susceptible *E. coli* clones (ST452, ST569, ST681, ST998) detected in the urogenital microbiome of healthy women, since they are poorly represented in public databases. Nevertheless, they have been previously reported as animal colonizers or from human infections in different continents (Figure 5), often associated with antibiotic resistance. For instance, *mcr*-1 positive ST452 or ESBL producing ST998 *E. coli* strains were previously isolated from animals, including domestic animals, i.e., dog or human UTI in five continents [64,65,66,67,68]. Similarly, ST681 strains have been detected worldwide in animals (wild boars, non-human primates) and among isolates causing human ExPEC infections, including UTI [69,70,71,72]. Furthermore, besides reports on UTI caused by ST569, this lineage could have possible food and environmental reservoirs, as, in the USA, ST569 *E. coli* was found in meat [73] and it was detected in wastewater in South Africa [74]. Mbanga et al. also demonstrated that a strain isolated from wastewater clustered together with previously isolated clinical ST998 *E. coli* isolated from a UTI patient in the same area [65].

Nevertheless, whether those strains possess the ability to persistently colonize the human urogenital tract, or if they are only transient microbiome members remains to be evaluated. Furthermore, one of the most fundamental questions is still unanswered, specifically, the circumstances that would trigger the development of infection by those colonizing strains [75].

The limitations of this study include the small sample size and the absence of fecal samples from the same individuals to assess concomitant gastrointestinal colonization; however, the in silico comparative genomic analysis performed showed a close relatedness of urinary and gastrointestinal strains. Using voided urine was also considered as a limitation, due to the possibility of collecting microbes that reside in the vaginal environment, but we demonstrated that a voided urine sample can provide distinct strains to those found in vagina. Furthermore, insights from this type of sample are valuable, as it is used in current UTI diagnostic practices.

## 5. Conclusions

In this study, we demonstrated that *E. coli* strains identified in the urogenital microbiome of healthy women frequently belong to international, pandemic, and occasionally antibiotic resistant B2 clones. Our data support the role of the urogenital tract as a source of ExPEC strains prone to cause UTI, which most probably originate from the gastrointestinal tract. Furthermore, we demonstrated that diverse *E. coli* lineages can be observed per individual and urogenital sample type, which should be considered in future studies focusing on *E. coli* screening. These findings are a hallmark to further understand the ecology of these clones and their distribution in human host reservoirs.

The influence of microbial communities, the host and environmental features in triggering the transition from colonization to infection is still unknown, but this subject deserves further investigation.

## Figures and Tables

**Table 1:** Phylogenetic origin and diversity of urinary (*n* = 10) and vaginal (*n* = 12) *E. coli* strains isolated from healthy women.

Donor	Origin	Strains	Phylogenetic Group	ExPEC	PFGE	WGS	MLST	Serotype
1	U	c1Ub_48	F	+	EC1	−	−	−
	VS	c1VSb_14	F	+	EC1	−	−	−
	U	c1Ub_56	B2	+	−	+	ST452	O81:H27
3	U	c3Ub_1	B2	+	EC2	+	ST681	O8:H10
	VS	c3VSb_22	B2	+	EC2	−	−	−
7	VS	c7VSa_62	D	−	−	−	−	−
10	U	c10Ua_105	F	+	EC3	−	−	−
	VS	c10VSa_39	F	+	EC3	−	−	−
11 *	U	c11Ua_88	D	−	−	−	−	−
	U	c11Ub_17	B2	+	EC4	+	ST1154 (ST73 complex)	O2:H1
	VS	c11VSb_7	B2	+	EC4	−	−	−
	VS	c11VSb_12	B2	+	EC5	+	ST998	O2:H6
12	VS	c12VSb_42	C	−	−	−	−	−
15	U	c15Ub_26	A	−	EC6	−	−	−
	VS	c15VSb_20	A	−	EC6	−	−	−
26	U	c26Ub_7	B2	+	EC7	+	ST131	O25:H4
	VS	c26VSb_8	B2	+	EC7	−	−	−
29 *	U	c29Ub_57	D	−	−	−	−	−
	VS	c29VSb_15	B2	+	EC8	+	ST140 (ST95 complex)	O2:H5
	VS	c29VSa_23	B2	−	EC9	+	ST569	O46:H31
31	U	c31Ua_56	E	−	−	−	−	−
	VS	c31VSa_9	B2	+	−	+	ST127	O6:H31

U-urine; VS- vaginal swab; ExPEC-extraintestinal pathogenic *E. coli*; PFGE-pulsed-field gel electrophoresis; WGS-whole genome sequencing; MLST-multilocus sequence typing. *-donors from whom *E. coli* was detected at 2 different sampling times; ‘a’ and ‘b’ in strain name denotes the sampling time.

**Table 2:** Detection and prevalence (%) of phylogenetic groups and 41 putative virulence genes among 22 unique *E. coli* strains per each sample type.

	Total (*n* = 22)	Urinary Isolates (*n* = 10)	Vaginal Isolates (*n* = 12)
**Phylogenetic group**			
A	2 (9)	1 (10)	1 (8)
B2	11 (50)	4 (40)	7 (58)
C	1 (5)	0	1 (8)
D	3 (14)	2 (20)	1 (8)
E	1 (5)	1 (10)	0
F	4 (18)	2 (20)	2 (17)
**Adhesins**			
*fimH*	20 (91)	9 (90)	11 (92)
*papAH*	3 (14)	2 (20)	1 (8)
*papC*	7 (32)	3 (30)	4 (33)
*papEF*	7 (32)	3 (30)	4 (33)
*papG* II, III	1 (5)	0	1 (8)
*papG allele* III	3 (14)	1 (10)	2 (17)
*sfa/focD*	6 (27)	2 (20)	4 (33)
*sfaS*	1 (5)	0	1 (8)
*focG*	6 (27)	2 (20)	4 (33)
*afa/draBC*	3 (14)	2 (20)	1 (8)
*iha*	7 (32)	4 (40)	3 (25)
*matB*	18 (82)	8 (80)	10 (83)
*yfcV*	15 (68)	6 (60)	9 (75)
**Toxins**			
*hlyA*	3 (14)	1 (10)	2 (17)
*cnf1*	3 (14)	1 (10)	2 (17)
*sat*	9 (41)	4 (40)	5 (42)
*tsh*	8 (36)	3 (30)	5 (42)
*vat*	5 (23)	1 (10)	4 (33)
*tosA*	4 (18)	2 (20)	2 (17)
**Siderophores**			
*fyuA*	14 (64)	8 (80)	6 (50)
*iutA*	8 (36)	5 (50)	3 (25)
*iroN*	8 (36)	3 (30)	5 (42)
*ireA*	3 (14)	1 (10)	2 (17)
**Capsule**			
*kpsMT* II	12 (55)	5 (50)	7 (58)
*kpsMT* III	2 (9)	1 (10)	1 (8)
*kpsMT* K1	5 (23)	1 (10)	4 (33)
*kpsMT* K5	6 (27)	3 (30)	3 (25)
**Protectins**			
*cvaC*	2 (9)	1 (10)	1 (8)
*traT*	12 (55)	6 (60)	6 (50)
*iss*	2 (9)	1 (10)	1 (8)
**Invasins**			
*ibeA*	3 (14)	0	3 (25)
*gimB*	1 (5)	0	1 (8)
**Miscellaneous**			
*usp*	13 (59)	5 (50)	8 (67)
*ompT*	18 (82)	8 (80)	10 (83)
PAI *(malX)*	12 (55)	7 (70)	5 (42)
*pafP*	15 (68)	6 (60)	9 (75)
*upaH*	11 (50)	4 (40)	7 (58)

There was no isolate positive for *papG* allele II, *bmaE*, *gafD*, *rfc*. None of the isolates tested had two copies of the *hlyA* operon.

## Data Availability

The Whole Genome Shotgun project of the isolates c1Ub_56_AE, c3Ub_1_AE, c11Ub_17_AN, c11VSb_12_AN, c13Ua_2_AN, c14Ub_22_AE, c26Ub_7_AN, c29VSa_23_AE, c29VSb_15_M, c31VSa_9_AN, c32Ub_19_M have been deposited at DDBJ/ENA/GenBank under the accession numbers JAECYT000000000, JAECYS000000000, JAECYR000000000, JAECYQ000000000, JAECYP000000000, JAECYO000000000, JAECYN000000000, JAECYM000000000, JAECYL000000000, JAECYK000000000, JAECYJ000000000, respectively.

## Supplementary Materials

The following are available online at https://www.mdpi.com/article/10.3390/microorganisms10010027/s1, Figure S1: Pulsed-field Gel Electrophoresis (PFGE) of selected *E. coli* isolates. Figure S2: Whole genome SNPs phylogenetic tree of 272 ST95 and ST140 (SLV 95) *E. coli* genomes of human origin. Figure S3: Whole genome SNPs phylogenetic tree of 217 ST73 and ST1154 (SLV 73) *E. coli* genomes of human origin.; Figure S4: Whole genome SNPs phylogenetic tree of 66 ST127 *E. coli* genomes of human origin. Table S1: List of donors, samples and isolates subjected to analysis in this study. Table S2: List of primers used for detection of ExPEC virulence genes. Table S3: Assemblies statistics and accession numbers per each novel strain used in this study (BioProject accession number PRJNA548360). Table S4: SNPs matrix for ST131 core genome.
