# Supplementary material for: The Darkest Place Is under the Candlestick-Healthy Urogenital Tract as a Source of Worldwide Disseminated Extraintestinal Pathogenic Escherichia coli Lineages
Source: Microorganisms. 2021 Dec 24;10(1):27. doi: 10.3390/microorganisms10010027 (PMC8778945; doi:10.3390/microorganisms10010027)
Supplement: Supplementary file 1 [file microorganisms-10-00027-s001.zip › Supplementary Figures S1-S4.pdf]

Supplementary material

The darkest place is under the candlestick - healthy urogenital tract as a source of worldwide disseminated extraintestinal pathogenic *Escherichia coli* lineages

Magdalena Ksiezarek[1,2], Angela Novais[1,2], *Luisa Peixe[1,2]

[1]UCIBIO – Applied Molecular Biosciences Unit, REQUIMTE, Faculty of Pharmacy, Department of Biological Sciences, Laboratory of Microbiology, University of Porto, 4050-313 Porto, Portugal

[2]Associate Laboratory i4HB - Institute for Health and Bioeconomy, Faculty of Pharmacy, University of Porto, 4050-313 Porto, Portugal

Supplementary Figure S1. Pulsed-field Gel Electrophoresis (PFGE) of selected E. coli isolates.

M - marker; **1** - 1Ub_83; **2** - 1VSb_14; **3** - 10Ua_105; **4** - 10VSa_39; **5** - 15Ub_26; **6** - 15VSb_20; **7** - 29VSa_23; **8** - 29VSb_15; **9** - 3Ub_1; **10** - 3VSb_22; **11** - 11Ub_17; **12** - 11VSb_7; **13** - 11VSb_12; **14** - 26Ub_7; **15** - 26VSb_8; **-** other isolates.

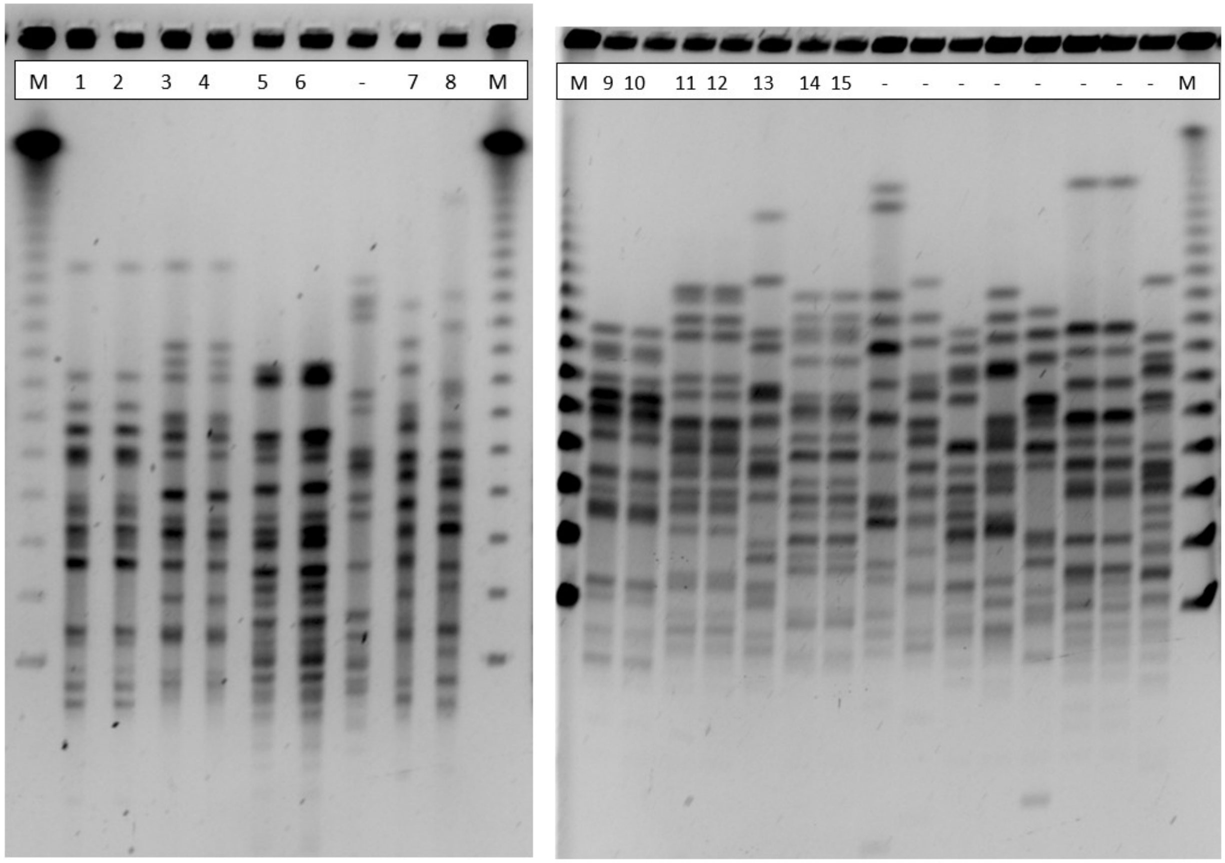

Supplementary Figure S2. Whole genome SNPs phylogenetic tree of 272 ST95 and ST140 (SLV 95) *E. coli* genomes of human origin. The alignment was performed using *E. coli* strain UTI89 as a reference. The metadata including host health status and strains origin is incorporated in the tree, as explained in the legend. The identifier of 1 strain from our urogenital collection is marked in red. The identifiers of 3 strains from Garretto et al. 2020 are marked in blue.

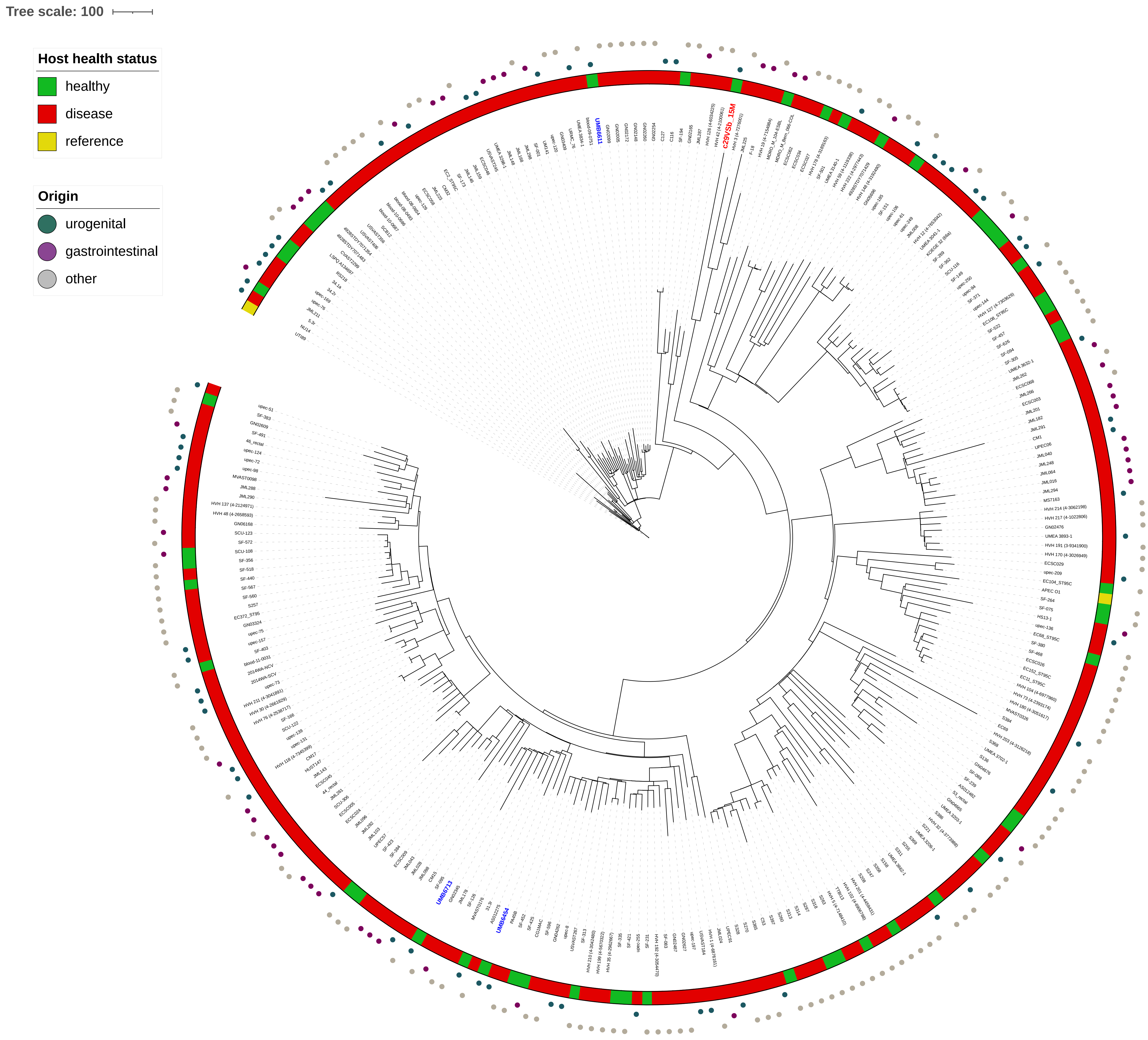
Tree scale: 100
Host health status
healthy
disease
reference
Origin
urogenital
gastrointestinal
other
c29VSb_15M
UMB6611
UMB6713
UMB6454

Supplementary Figure S3. Whole genome SNPs phylogenetic tree of 217 ST73 and ST1154 (SLV 73) *E. coli* genomes of human origin. The alignment was performed using *E. coli* strain CFT073 as a reference. The metadata including host health status and strains origin is incorporated in the tree, as explained in the legend. The identifiers of 2 strain from our urogenital collection are marked in red.

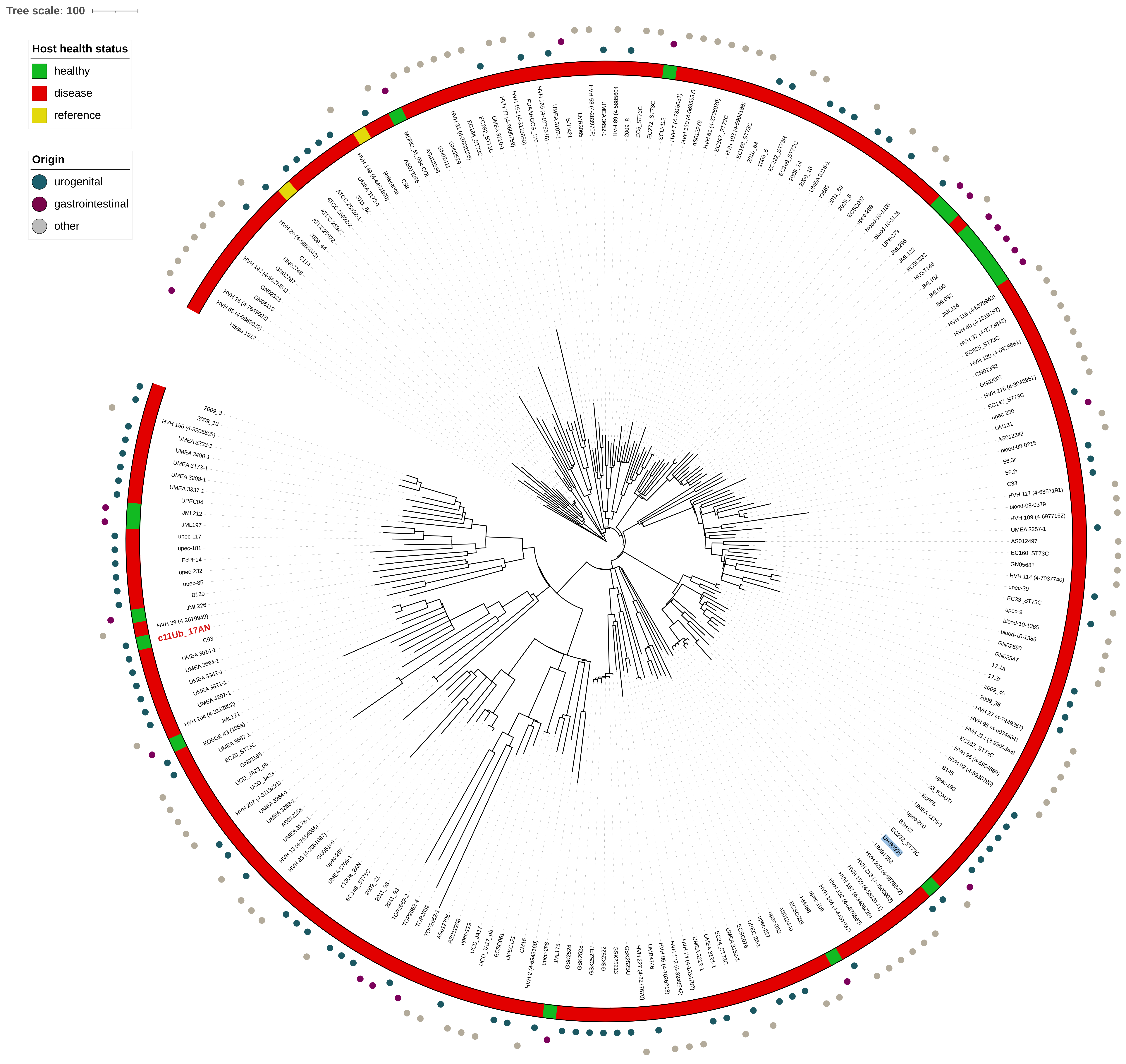
Tree scale: 100
Host health status
healthy
disease
reference
Origin
urogenital
gastrointestinal
other
c11Ub_17AN

Supplementary Figure S4. Whole genome SNPs phylogenetic tree of 66 ST127 *E. coli* genomes of human origin. The alignment was performed using *E. coli* strain 536 as a reference. The metadata including host health status and strains origin is incorporated in the tree, as explained in the legend. The identifier of strain from our urogenital collection is highlighted in green.

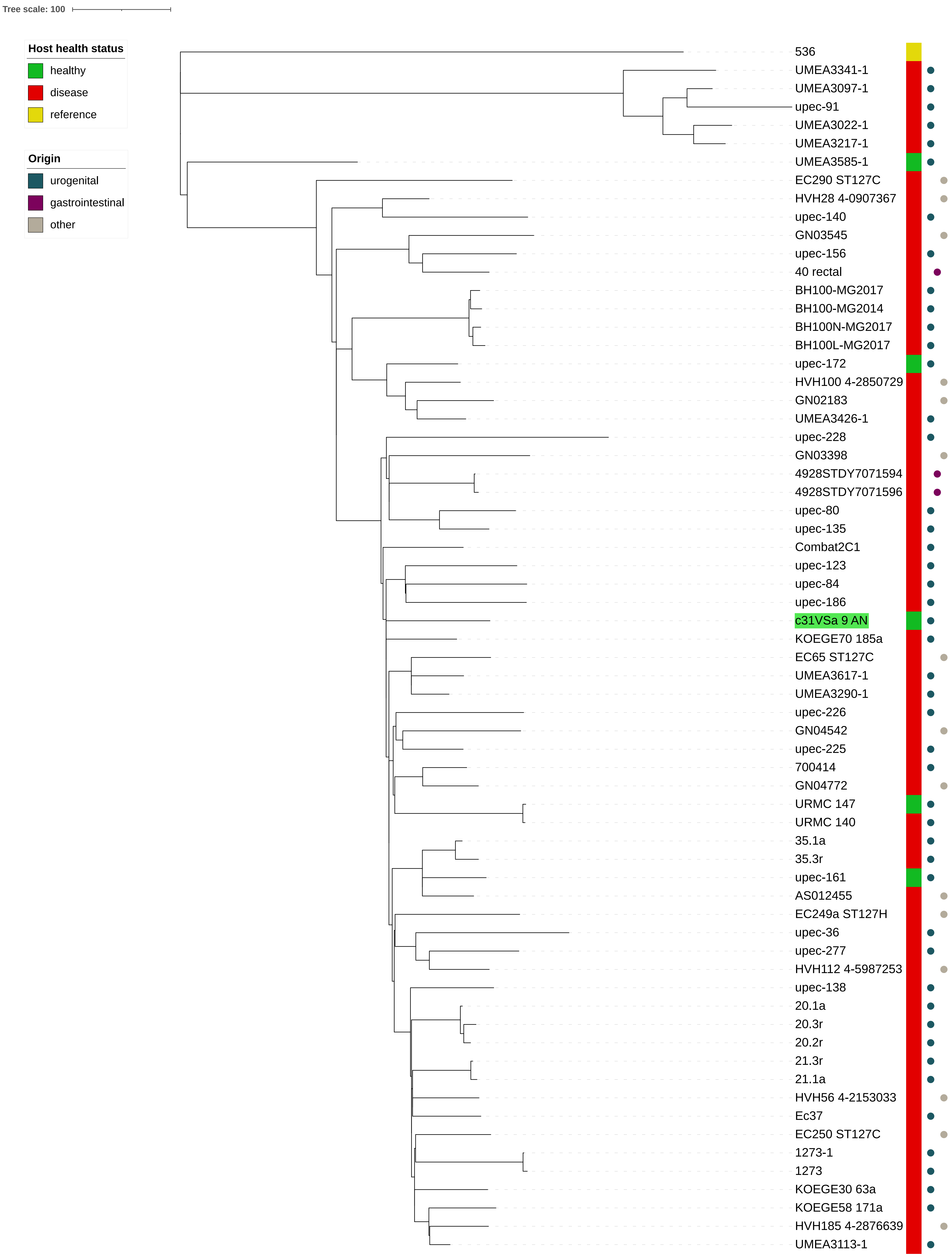
Tree scale: 100
Host health status
healthy
disease
reference
Origin
urogenital
gastrointestinal
other
536
UMEA3341-1
UMEA3097-1
upec-91
UMEA3022-1
UMEA3217-1
UMEA3585-1
EC290 ST127C
HVH28 4-0907367
upec-140
GN03545
upec-156
40 rectal
BH100-MG2017
BH100-MG2014
BH100N-MG2017
BH100L-MG2017
upec-172
HVH100 4-2850729
GN02183
UMEA3426-1
upec-228
GN03398
4928STDY7071594
4928STDY7071596
upec-80
upec-135
Combat2C1
upec-123
upec-84
upec-186
c31VSa 9 AN
KOEGE70 185a
EC65 ST127C
UMEA3617-1
UMEA3290-1
upec-226
GN04542
upec-225
700414
GN04772
URMC 147
URMC 140
35.1a
35.3r
upec-161
AS012455
EC249a ST127H
upec-36
upec-277
HVH112 4-5987253
upec-138
20.1a
20.3r
20.2r
21.3r
21.1a
HVH56 4-2153033
Ec37
EC250 ST127C
1273-1
1273
KOEGE30 63a
KOEGE58 171a
HVH185 4-2876639
UMEA3113-1
